# Modelling the links between farm characteristics, respiratory health and pig production traits

**DOI:** 10.1038/s41598-021-93027-9

**Published:** 2021-07-02

**Authors:** H. Gray, M. Friel, C. Goold, R. P. Smith, S. M. Williamson, L. M. Collins

**Affiliations:** 1 School of Biology, Faculty of Biological Sciences, University of Leeds, Leeds, UK; 2 Animal and Plant Health Agency (APHA), Weybridge, UK; 3 APHA, Rougham Hill, Bury St Edmunds, Suffolk, UK

**Keywords:** Zoology, Animal physiology

## Abstract

Sustainable livestock production requires links between farm characteristics, animal performance and animal health to be recognised and understood. In the pig industry, respiratory disease is prevalent, and has negative health, welfare and economic consequences. We used national-level carcass inspection data from the Food Standards Agency to identify associations between pig respiratory disease, farm characteristics (housing type and number of source farms), and pig performance (mortality, average daily weight gain, back fat and carcass weight) from 49 all in/all out grow-to-finish farms. We took a confirmatory approach by pre-registering our hypotheses and used Bayesian multi-level modelling to quantify the uncertainty in our estimates. The study findings showed that acquiring growing pigs from multiple sources was associated with higher respiratory condition prevalence. Higher prevalence of respiratory conditions was linked with higher mortality, and lower average daily weight gain, back fat and pig carcass weight. Our results support previous literature using a range of data sources. In conclusion, we find that meat inspection data are more valuable at a finer resolution than has been previously indicated and could be a useful tool in monitoring batch-level pig health in the future.

## Introduction

An increasing human population requires that global agricultural outputs increase by at least 50% by 2050^1–3^, including arable, horticultural and animal products. Not only will there be more people to feed, but for some animal products (pork and poultry), there are trends of increased consumption^4^. Per capita consumption of pork increased from 2012 to 2018, and pork was the most consumed meat product per capita in the European Union, China, Korea and Vietnam in 2018^4^. In the UK, the annual consumption of pork increased by 1.5 kg per person from 2008 to 2018^5^.

An increasing demand for pork, paired with concerns for the environmental pressures caused by agriculture^3^, means that farmers must provide larger quantities of sustainably-produced meat. Understanding how to respond to this demand can be aided by identifying relevant links between good animal health, efficient production and farm infrastructure. Evidencing these links helps farmers make appropriate changes to produce healthy, productive animals under optimal conditions.

In the United Kingdom, health and performance data collection in the pig sector is commonly practised but not in a standardised manner. Data exist at different scales and resolutions and often in multiple, unlinked datasets with different owners. One of the largest national datasets is collected by the Food Standards Agency (FSA), which conducts ante- and post-mortem inspections on every pig submitted for slaughter in England to ensure it is fit for human consumption. Finer-scale health and welfare records are also maintained by farmers and pig companies, as well as by food assurance schemes (e.g. Red Tractor, RSPCA Freedom Food and Soil Association). Production and farm characteristics data may be recorded by pig producers at both batch and farm level to monitor production efficiency and to track the effects of farm characteristics decisions and disease control interventions. Integrating subsets of these locally- and nationally-held data sources can help to clarify the associations between certain health conditions, farm characteristics decisions, and production outcomes.

Respiratory disease is of particular importance in the pig industry as it presents a major health and welfare challenge, resulting in economic losses for producers^6–8^. Respiratory disease is often a complex, multifactorial syndrome resulting from pathogens—viral, bacterial and parasitic—acting singly or in combination with the extent and severity of disease influenced by environmental/farm characteristics (e.g. temperature, humidity, hygiene) and host factors (e.g. pathogen and immune status). Depending on the severity, respiratory disease may manifest in overt clinical signs and mortality, or be subclinical, with the adverse effects noticed through poorer than expected performance. In either situation, gross lesions of the respiratory tract detected at the abattoir provide a measure of the extent of disease.

Studies have attempted to quantify respiratory prevalence at differing resolutions, both on farm and at the abattoir. Controlled studies examining the on-farm presentation or post-mortem characteristics of specific respiratory conditions often involve labour-intensive data collection at slaughter and/or on-farm, including comprehensive diagnostic testing and serological testing to determine infection and previous exposure to respiratory pathogens. These more targeted approaches provide useful insights into disease on individual farms or within batches of pigs but typically are costly and involve relatively few farms, smaller numbers of pigs, and do not give an overarching view of the associations between farm characteristics, disease and production. By contrast, a small number of studies have investigated national datasets to validate their surveillance potential, to analyse temporal prevalence patterns and/or to link slaughter data to on-farm risk factors and production outputs (Real Welfare Scheme^9^; British Pig Health Scheme/BPEX Health Scheme^10–14^; Wholesome Pigs Scotland^10,11^; Pig Regen Ltd. health and welfare checks^11^). These datasets provide more standardised and detailed scoring of conditions in samples of slaughtered pigs, but they do not have the coverage of assessing every slaughtered pig.

Previous studies have investigated the associations between respiratory conditions and farm characteristics factors^14–23^, as well as between respiratory conditions and production traits^6,12,24–28^. Notable findings indicate that housing features, such as natural ventilation^17,29^ and lack of disinfection^14,16^, can increase the risk of respiratory conditions and that, in turn, respiratory conditions can have a negative impact on production traits such as average daily weight gain^6^ and carcass weight^26^.

Despite previous studies linking respiratory disease to production traits and farm characteristics factors, the majority of those studies have been exploratory and, thus, their results are correlational rather than causal. For example, previous studies have included multiple independent farm-level variables (in some cases more than the number of data points; e.g.^16^) and relatively low sample sizes. These characteristics of exploratory research risk increasing false positives^30^ and require verification through replication attempts and confirmatory research^31^. Determining causal associations requires confirmatory approaches testing a priori hypothesised relationships between pig health, production traits and farm characteristics, including representation of uncertainty in these relationships.

**Figure 1:**
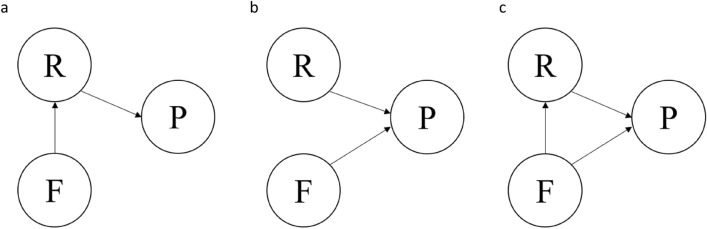
Conceptual diagrams for (**a**) Model 1, (**b**) Model 2 and (**c**) Model 3, where F represents farm characteristics, R represents respiratory prevalence and P represents production outcomes. The arrows indicate the direction of the relationships.

In this study, we examined the links between specific characteristics of farm infrastructure and farm characteristics, respiratory conditions and production traits using routinely collected production and meat inspection data—the Collection and Communication of Inspection Results (CCIR) data (Food Standards Agency). Although meat inspection data has been criticised for being of low resolution for surveillance purposes^32,33^, it provides the largest dataset on livestock health conditions at slaughter in the UK and has been proposed as a caveated method for monitoring animal welfare^34^. We guarded our investigation from spurious findings by (i) adopting a confirmatory approach by testing a priori hypotheses based on theory; (ii) using Bayesian multi-level modelling and model selection using k-fold cross-validation to analyse all data sources within a single model and estimate parameter uncertainty; and (iii) pre-registering our hypotheses and methods on the Open Science Framework (https://osf.io/hu78g). Specifically, we tested the following hypotheses:

Global hypotheses (Fig. 1):

1. Farm characteristics impact respiratory condition prevalence, which then influences production outcomes.
2. Both farm characteristics and respiratory condition prevalence influence production outcomes, but there is no direct effect of farm characteristics on respiratory conditions.
3. Both farm characteristics and respiratory condition prevalence influence production outcomes, and farm characteristics factors have an impact on respiratory condition prevalence.

Specific hypotheses:

1. Disinfection: Disinfecting a room/building between batches of pigs will be associated with lower prevalence of respiratory conditions compared with when rooms are not disinfected between batches^14,16,35^.
2. Batch source: Acquiring pigs from one source farm will be associated with lower prevalence of respiratory conditions compared with sourcing pigs from multiple farms^14,17^.
3. Ventilation: Assisted ventilation (as opposed to passive/natural ventilation such as a window) will be associated with lower prevalence of respiratory conditions compared with natural ventilation^15–17,29^.
4. Housing: Housing systems containing straw will be associated with higher prevalence of respiratory conditions than housing systems not containing straw^22,36^. Straw systems may have less control over the internal environment (e.g. curtains on barns versus an indoor thermostat system) and straw can contribute to dust which can have adverse respiratory effects.
5. Time: A higher average number of days spent on farm for finisher pigs will be associated with higher prevalence of respiratory conditions, compared to fewer days spent on farm.
6. A reduction in the prevalence of respiratory conditions will be associated with more favourable production traits: heavier deadweight, higher average daily weight gain, lower levels of finisher mortality, and more optimal back fat scores.

## Methods

This study was approved by the Biological Sciences Faculty Research Ethics Committee, University of Leeds (Reference: LTSBIO-006).

### Datasets

#### Health data

Measures of respiratory disease were calculated using data provided by the FSA via their CCIR system. These data are collected at the abattoir by official veterinarians (ante-mortem) and meat health inspectors (post-mortem). CCIR is a compulsory system used to record presence or absence of specific conditions that can lead to partial or total rejection of a carcass. For each slaughter batch of pigs, the number of pigs with each condition observed is recorded. A slaughter batch refers to a delivery of pigs to the abattoir from the same farm, slaughtered on the same day. We were provided with CCIR data from September 2009–December 2015. A subset of the CCIR data (hereafter termed ‘health data’) were used to calculate prevalence of respiratory conditions for each production batch of pigs (described below). Ante- and post-mortem data were combined and respiratory conditions were regarded as any of the following reported conditions: abnormal breathing rate/depth; abnormal respiratory signs; coughing; pericarditis; pleurisy; pneumonia; respiratory; rhinitis; or twisted snout. If one pig had more than one condition (e.g. pneumonia and abnormal breathing) this would be recorded at batch level as two counts as conditions are not attributed to individual pigs.

#### Farm characteristics data

A random sample of 25 pig companies were directly contacted via email and invited to participate in the study. Six companies (24%) agreed to participate, four in England and two in Northern Ireland. However, it was not possible to gain CCIR data from Northern Ireland and therefore the data from these companies were excluded from the current study. Three out of the four English companies agreed to provide both farm characteristics and production data and a questionnaire was sent to the production managers of these companies. The data were gathered through an electronic (Microsoft Word document) questionnaire (not part of the pre-registration). The questionnaire was completed for 105 farms. Twenty-eight were self-completed by production managers of two companies. The remaining 77 were completed by a researcher (MF), through interviews with the managing director and being given access to the data required to complete the questionnaires.

The full questionnaire can be found in supplementary materials (S1), but the questions of interest for this study pertained to: disinfection of buildings, housing type, number of source farms from which pigs were acquired, and ventilation type. Disinfection was a binary (yes/no) category depending on whether disinfectant was used in buildings between batches of pigs. Finisher housing type consisted of four categories: straw yards, slatted, kennels or mixed (where a mixture of housing types were used). Ventilation was a binary variable, with the options being natural or assisted (i.e. the use of mechanical fans) ventilation. Batch source was a categorical variable with options of acquiring pigs from one, two, three, or more than three sources. Although genetic information is an important variable in explaining production parameters, this was not requested due to its commercial sensitivity and its variation within farms through time.

#### Production data

Finishing pig production data, at the batch level, were provided on a voluntary basis by the participating companies, with the variables of interest shown in Table 1. Only pigs slaughtered from growing herds (no breeding pigs) and all-in/all-out batches were used. In an all-in/all-out system, production batches enter holdings sequentially with a break in production between batches, allowing consecutive batches to be distinguishable within the health data. By contrast, in a continuous system, batches overlap and slaughter batches may be made up of pigs from several production batches. Attributing conditions in the health data to different production batches in a continuous system is particularly challenging without individual-level tagging and tracing, which is not widely practiced commercially.

**Table 1:** Production batch data variable descriptions and an indication of whether these were used for data matching or data analysis.

Variable	Explanation	Use
Slapmark	Slapmark for farm identification. This is used for matching to other datasets	Matching
Pig entry date	Date which pigs were placed on the farm	Matching
Mortality	Number of pigs that died between arrival and slaughter	Analysis
Average days on site	Average number of days between arrival and slaughter	Analysis
Total placed	Number of pigs placed on the farm to make up a batch	Analysis
Total sold	Number of pigs sent to slaughter	Matching
Average daily weight gain	Average kg of weight gained per pig from placement on farm to slaughter	Analysis
Average deadweight	Average carcass weight for the production batch	Analysis
Average P2	Average back fat measured on the carcass by probe in the abattoir	Analysis

### Data cleaning and matching

**Figure 2:**
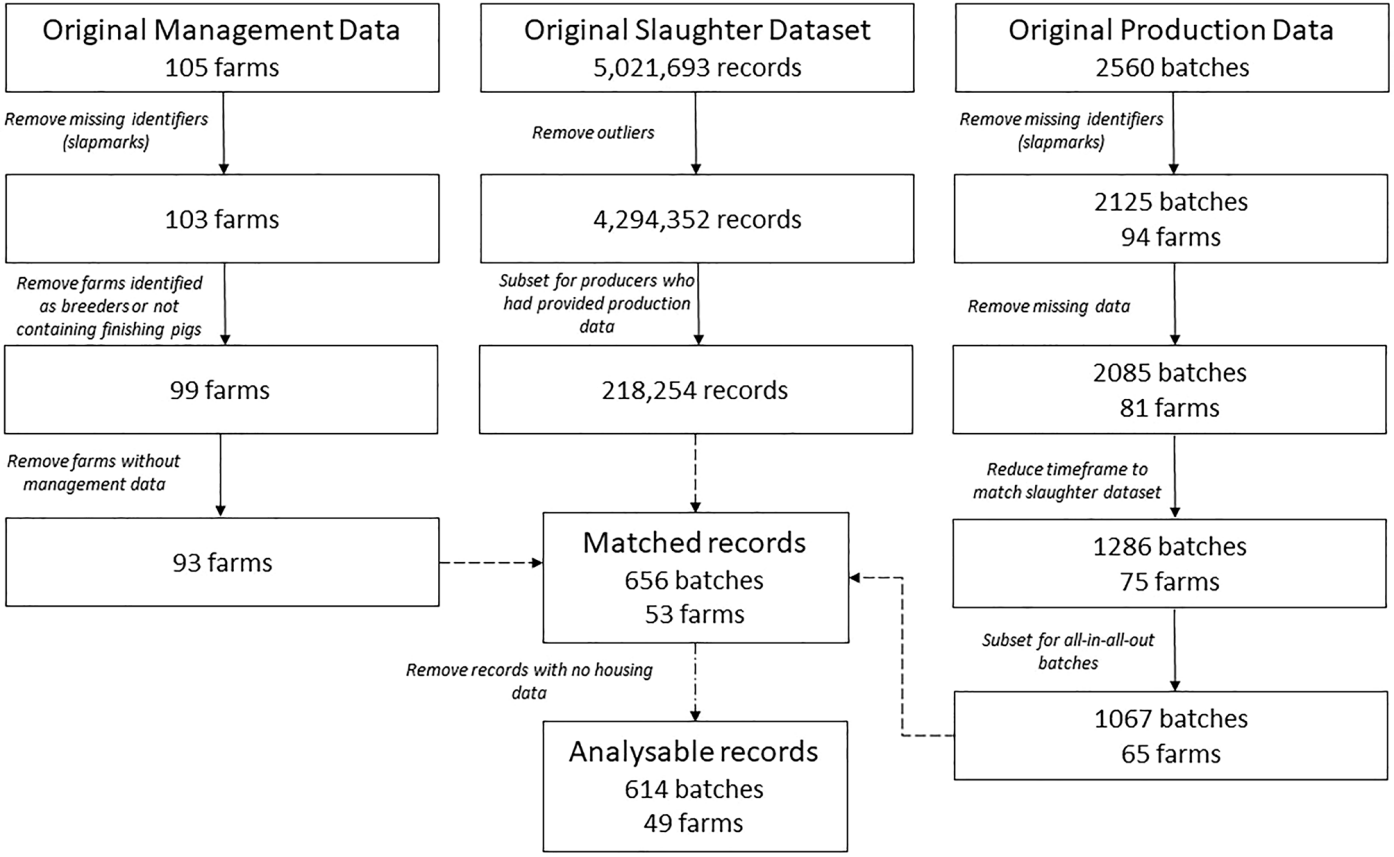
Data cleaning process showing how farm characteristics data, slaughter data, and production data were filtered and depicting the resulting inclusions at each stage (solid arrows). Matching between the three datasets is denoted by the dashed arrows.

Due to the different resolutions and formats, datasets needed to be cleaned before being matched. Briefly, outliers were removed from health data as the dataset was found to contain erroneous entries, which did not match the nationally reported slaughter numbers from the Agriculture and Horticulture Development Board (AHDB; levy board). Including these erroneous entries would have resulted in an overcounting of respiratory conditions. Any entry containing > 10, 000 pigs slaughtered per day per abattoir was removed as this is not logistically possible and was therefore considered as an error. Production data were cleaned to remove records of farms with no unique identifiers as these could not be matched to slaughter health data. Farm characteristics data were retained if the farm provided a unique identifier and provided data for at least one category of interest (disinfection, ventilation, housing and number of source batches). Farms were removed from the dataset if they were not classified as a finishing farm (this resulted in three breeding farms and one wean-to-30 kg farm being excluded). Figure 2 depicts the cleaning and matching process and the exclusions at each stage.

Farm characteristics data and production data were matched by using unique slapmarks—the herd specific alpha-numeric code that is applied as a tattoo to each pig before slaughter to identify its farm of origin. Health data and production data were matched using slapmarks and estimated slaughter date ranges, to ensure attribution to the correct batch of pigs from a farm. For example, for one production batch of pigs from one farm, we calculated the slaughter date range as − 35 and + 28 days (see Table S1 for sensitivity analysis) from the average slaughter date to give a minimum and maximum slaughter date. The slapmark was searched for in the health dataset and, if found, the slaughter date was checked to see if it fell within the estimated range. If both the slaughter date and slapmark corresponded, the number of pigs slaughtered and the number of respiratory conditions found at slaughter were both summed separately to give a total number of pigs and a total number of respiratory conditions for each batch, respectively. Matches for a production batch of pigs were deemed correct if the number of pigs returned from the CCIR data was within 230 of the number reported as sold. Two hundred and thirty was chosen as an appropriate cut off as this represents one delivery of pigs to the abattoir. A total of 656 batches from 53 farms were matched for production, CCIR and farm characteristics data.

### Data processing

Following data matching and cleaning, the farm characteristics predictor variables (batch source, housing, ventilation and disinfection) were checked for frequency in the dataset and collinearity (see pre-registration). Ventilation and disinfection showed complete collinearity; only 20 (3%) batches had both assisted ventilation and were disinfected. The remaining 636 batches had both natural ventilation and were not disinfected. There was not enough variation in these predictors to use in the planned analysis, so they were omitted from further analysis. Four producers did not provide data on housing. Given that housing and batch source were now the only farm characteristics being used as predictors, these four farms were removed from the analysis. This left a total of 49 farms with 614 batches of pigs for analysis, equating to 836,093 pigs sold to slaughter. Forty-six of the farms were from one company and three of the farms from a second company. The variables of interest were then transformed for analysis (see Table 2).

**Table 2:** Pig batch-level variables used in analyses and transformations for analysis.

Variable	Type	Transformation
Respiratory cases	Count	Mean-centred
Respiratory prevalence	Continuous	Mean-centred
Average daily weight gain	Continuous	Mean-centred
Batch source	Categorical	Sum-to-zero contrasts
Housing type	Categorical	Sum-to-zero contrasts
Batch size	Continuous	Mean-centred and scaled by 100 pigs
Time (days on farm)	Continuous	Mean-centred and scaled by 7 days

### Statistical methods

The methods for analysis were pre-registered, with full details, amendments and code available at https://osf.io/hu78g. All data cleaning, processing and analysis was conducted in R v. 3.6.1^37^. We tested three Bayesian multi-level model structures according to the different global hypotheses between respiratory conditions, farm characteristics, and production traits. The production outcomes were modelled separately because different global hypotheses may be better suited to different production traits. Weakly informative priors were used on all predictor regression coefficients (normal distributions with mean 0 and SD 1; see Figure S1) to mitigate against large, unlikely effect sizes and to aid computation. Data for P2 were missing for 126 batches (20.5%). These were treated as missing at random and were imputed within the model.

Models were computed using the Stan programming language^38^ via the brms package (version 2.12.0^39^), which estimates parameters using Hamiltonian Monte Carlo. Four Markov chains were run, each with a warm-up period of 2500 iterations and 2500 iterations used for sampling. Thinning was set to 1. Convergence was checked using the Gelman–Rubin statistic with convergence indicated by values close to 1 and less than 1.05. Model comparisons were conducted using K-fold cross validation (in the brms package), whereby the model with lowest information criteria score (defined as − 2 times the expected log predictive density; see^40^) indicates the best fit. The number of K-folds was set to 10. Model parameters were summarised by the mean and 95% highest density interval (HDI; the 95% most likely values in the distribution). Significance was inferred when the highest density interval did not contain zero.

#### Model 1

This model tests the hypothesis that farm characteristics (shown by F in the conceptual diagram; Fig. 2) impacts respiratory rate (R) which then influences the production outcomes (P).

$$\begin{aligned} &R_{F[i]} \sim Poisson (\lambda_{1F[i]})\\ &log(\lambda_{1F[i]}) = \alpha_1 + \beta_{H}H_{F}+ \beta_{B}B_{F} + \beta_{T_{1}}T_{F[i]} + \beta_{S_{1}}S_{F[i]} + r_{1F} \\ &DW_{F[i]} \sim Normal^+(\mu_{1F[i]}, \sigma_1)\\ &\mu_{1F[i]} = \alpha_2 + \beta_{R_{1}}R_{F[i]} + r_{2F} \\ &M_{F[i]} \sim NegativeBinomial(\lambda_{2F[i]})\\ &log(\lambda_{2F[i]}) = \alpha_3 + \beta_{R_{2}}R_{F[i]} + \beta_{T_{2}}T_{F[i]} + \beta_{S_{2}}S_{F[i]} + r_{3F} \\ &P2_{F[i]} \sim Normal^+(\mu_{2F[i]}, \sigma_2)\\ &\mu_{2F[i]} = \alpha_4 + \beta_{R_{3}}R_{F[i]} + \beta_{G_{1}}G_{[i]} + r_{4F} \\ &ADWG_{F[i]} \sim Normal^+(\mu_{3F[i]}, \sigma_3)\\ &\mu_{3F[i]} = \alpha_5 + \beta_{R_{4}}R_{F[i]} +\beta_{T_{3}}T_{F[i]}+ r_{5F} \end{aligned}$$

Counts of respiratory conditions (R) for each batch (*i*) within a farm (*F*) are Poisson distributed, with rate λ_1_. The respiratory rate is a function of an intercept (α_1_), farm characteristics of housing (*β*_*H*_*H*_*F*_) and batch source (*β*_*B*_*B*_*F*_), the number of days on farm (*β*_*T1*_*T*_*F[i*]_), the batch size (*β*_*S1*_*S*_*F[i]*_) and a random intercept for each farm (*r*_*1F*_). Average days on farm is included because we assumed that the longer the pigs are on farm, the higher chance there is of contracting a respiratory condition. Batch size is included as respiratory conditions are expressed as count data and are therefore affected by the number of pigs in a batch.

Average deadweight (DW) for each batch (*i*) within a farm (*F*) are normally distributed with mean (µ_1_) and standard deviation (σ_1_). The mean is a function of an intercept (*α*_*2*_), the effect of respiratory prevalence (*β*_*R1*_*R*_*F*[*i*]_: counts of respiratory conditions divided by the production batch size) and a random intercept for each farm (*r*_*2F*_).

Mortality (M) for each batch (*i*) within a farm (*F*) is Poisson distributed with rate λ_2_. The rate is a function of an intercept (*α*_*3*_), the effect of respiratory prevalence (*β*_*R2*_*R*_*F*[*i*]_), the average number of days on the farm (*β*_*T2*_*T*_*F*[*i*]_), the batch size (*β*_*S2*_*S*_*F*[*i*]_) and a random intercept for each farm (*r*_*3F*_). Average days on farm is included as the age of entry influences the period of time that the mortality rate is recorded and mortality rates may also vary with age; batches of pigs entering the farm at an older age (with fewer days on site) will tend to have lower mortality recorded than those entering as younger pigs. Batch size at entry is included as mortality is expressed as count data and is therefore affected by the number of pigs in a batch.

Average back fat (P2) is measured on the carcass by probe in the abattoir for each batch (*i*) within a farm (*F*) and is normally distributed with mean (µ_2_) and standard deviation (σ_2_). The mean is a function of an intercept (*α*_*4*_), the effect of respiratory prevalence (*β*_*R3*_*R*_*F*[*i*]_), the effect of average daily weight gain (*β*_*G1*_*G*_*F*[*i*]_: a measure of growth, average grams gained per day) and a random intercept for each farm (*r*_*4F*_).

Average daily weight gain (ADWG) for each batch (*i*) within a farm (*F*) is normally distributed with mean (*µ*_*3*_) and standard deviation (*σ*_*3*_). The mean is a function of an intercept (*α*_*5*_), the effect of respiratory prevalence (*β*_*R4*_*R*_*F*[*i*]_), the average days on the farm (*Β*_*T3*_*T*_*F*[*i*]_) and a random intercept for each farm (*r*_*5F*_). Average days on farm is included as average daily weight gain is time-dependent. For example, pigs entering the farm at a younger age consume less feed and may have a lower average daily weight gain for their time on farm compared with those animals entering at an older age.

#### Model 2

Model two tests the hypothesis that both farm characteristics and respiratory rate influence production outcomes, but there is no direct effect of farm characteristics on respiratory conditions.

$$\begin{aligned} &R_{F[i]} \sim Poisson (\lambda_{1F[i]})\\ &log(\lambda_{1F[i]}) = \alpha_1 + \beta_{T_{1}}T_{F[i]} + \beta_{S_{1}}S_{F[i]} + r_{1F} \\ &DW_{F[i]} \sim Normal^+(\mu_{1F[i]}, \sigma_1)\\ &\mu_{1F[i]} = \alpha_2 + \beta_{H_{1}}H_{F}+ \beta_{B_{1}}B_{F}+ \beta_{R_{1}}R_{F[i]} + r_{2F} \\ &M_{F[i]} \sim NegativeBinomial(\lambda_{2F[i]})\\ &log(\lambda_{2F[i]}) = \alpha_3 + \beta_{H_{2}}H_{F}+ \beta_{B_{2}}B_{F}+ \beta_{R_{2}}R_{F[i]} + \beta_{T_{2}}T_{F[i]} + \beta_{S_{2}}S_{F[i]} + r_{3F} \\ &P2_{F[i]} \sim Normal^+(\mu_{2F[i]}, \sigma_2)\\ &\mu_{2F[i]} = \alpha_4 + \beta_{H_{3}}H_{F}+ \beta_{B_{3}}B_{F}+ \beta_{R_{3}}R_{F[i]}+ \beta_{G_{1}}G_{[i]} + r_{4F} \\ &ADWG_{F[i]} \sim Normal^+(\mu_{3F[i]}, \sigma_3)\\ &\mu_{3F[i]} = \alpha_5 + \beta_{H_{4}}H_{F}+ \beta_{B_{4}}B_{F}+ \beta_{R_{4}}R_{F[i]}+ \beta_{T_{3}}T_{F[i]} + r_{5F[i]} \end{aligned}$$

Respiratory counts are now modelled as a function of only the number of days on farm (*β*_*T1*_*T*_*F*[*i*]_), the batch size (*β*_*S1*_*S*_*F*[*i*]_), and a random intercept for each farm (*r*_*1F*_). All production outcome variables are modelled as in Model 1, with the addition that in Model 2 each has the farm characteristics in the deterministic part of the equation to reflect the direct link between farm characteristics and production in the model schematic.

#### Model 3

Model 3 is a combination of Models 1 and 2. This tests the hypothesis that both farm characteristics and respiratory rates influence production outcomes, as well as farm characteristics affecting respiratory rates.

$$\begin{aligned} &R_{F[i]} \sim Poisson (\lambda_{1F[i]})\\ &log(\lambda_{1F[i]}) = \alpha_1 + \beta_{H_{1}}H_{F}+ \beta_{B_{1}}B_{F} + \beta_{T_{1}}T_{F[i]} + \beta_{S_{1}}S_{F[i]} + r_{1F} \\ &DW_{F[i]} \sim Normal^+(\mu_{1F[i]}, \sigma_1)\\ &\mu_{1F[i]} = \alpha_2 + \beta_{H_{2}}H_{F}+ \beta_{B_{2}}B_{F[i]}+ \beta_{R_{1}}R_{F[i]} + r_{2F}\\ &M_{F[i]} \sim NagetiveBinomial(\lambda_{2F[i]})\\ &log(\lambda_{2F[i]}) = \alpha_3 + \beta_{H_{3}}H_{F}+ \beta_{B_{3}}B_{F}+ \beta_{R_{2}}R_{F[i]} + \beta_{T_{2}}T_{F[i]} + \beta_{S_{2}}S_{F[i]}+ r_{3F} \\ &P2_{F[i]} \sim Normal^+(\mu_{2F[i]}, \sigma_2)\\ &\mu_{2F[i]} = \alpha_4 + \beta_{H_{4}}H_{F}+ \beta_{B_{4}}B_{F[i]}+ \beta_{R_{3}}R_{F[i]} + \beta_{G_{1}}G_{[i]} + r_{4F} \\ &ADWG_{F[i]} \sim Normal^+(\mu_{3F[i]}, \sigma_3)\\ &\mu_{3F[i]} = \alpha_5 + \beta_{H_{5}}H_{F}+ \beta_{B_{5}}B_{F[i]}+ \beta_{R_{4}}R_{F[i]} + \beta_{T_{3}}T_{F[i]} + r_{5F}\\ \end{aligned}$$

Respiratory rates are modelled as in Model 1 and all production variables (deadweight, mortality, back fat and average daily weight gain) are modelled as in Model 2. Note here that farm characteristics are included in the deterministic parts of both the respiratory and production equations as farm characteristics now has two links in the conceptual diagram.

## Results

The final dataset contained pigs from 614 batches from 49 farms. Mean batch size was 1362 pigs (sd = 841; min = 116; max = 4696), mean start weight was 35.26 kg (sd = 3.92; min = 19.65; max = 77.54) and the mean days spent on the farm was 94 (sd = 10.15; min = 50; max = 129). Three farms used slats, nine used straw yards, 13 used mixed housing and 24 used kennels. All farms using kennels provided straw. Batch sources were as follows: (i) one source (10 farms); (ii) two sources (20 farms); (iii) three sources (10 farms); (iv) more than three sources (9 farms).

### Model fits

Model 1 was the best fit (had the best out of sample predictive accuracy) for mortality (K-fold information criteria: model 1 = 38,532; model 2 = 38,684; model 3 = 38,946) and average daily weight gain outcomes (K-fold information criteria: model 1 = 39,951; model 2 = 40,145; model 3 = 40,111), meaning these traits are likely to be affected by farm characteristics via the effect of farm characteristics on respiratory conditions. Model 3 was the best fit for deadweight (K-fold information criteria: model 1 = 37,538; model 2 = 37,401; model 3 = 36,655) and P2 (model 1 = 27,837; model 2 = 28,102; model 3 = 27,804), meaning these traits are influenced by farm characteristics directly, as well as indirectly through farm characteristics affecting respiratory rates.

### Respiratory conditions

**Figure 3:**
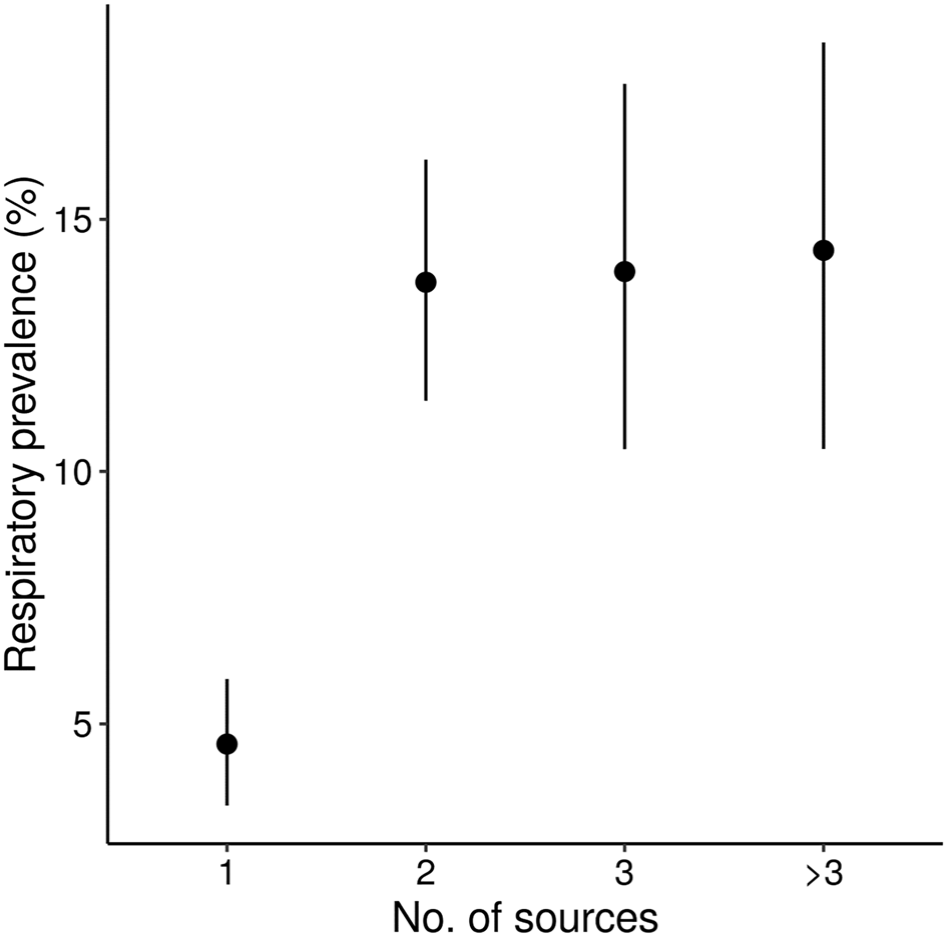
The effect of the number of source farms from which pigs are acquired on the respiratory prevalence at slaughter. Respiratory prevalence was calculated from the counts of respiratory conditions and the total pigs in the batch. Circles denote the mean posterior estimates and vertical line segments show their 95% highest density intervals.

**Figure 4:**
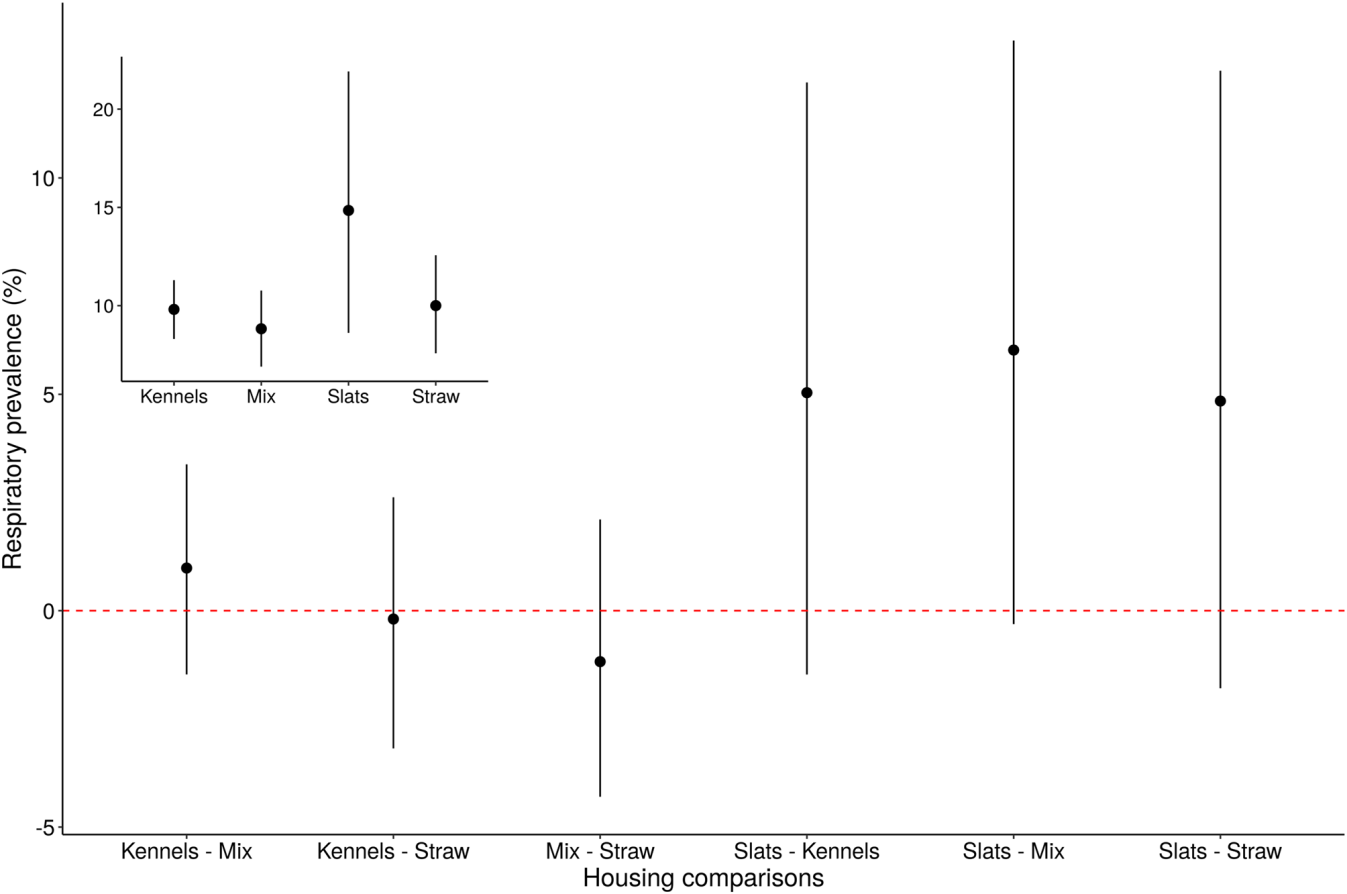
The difference in respiratory prevalence as a result of comparing the different housing types. Circles denote the mean posterior estimates and vertical line segments show their 95% highest density intervals. The horizontal dashed line indicates a difference of zero. The inset shows the predicted counts of respiratory conditions for each housing types. Respiratory prevalence was calculated from the counts of respiratory conditions and the total pigs in the batch. Kennels = kennelled housing; Mix = mixed housing (combinations of different housing types on one farm); Straw = straw yards and slats = slatted flooring.

The results presented in this section are model estimates and summary statistics, and all raw data are available in the supplementary material (Figure S1). Given that respiratory conditions were modelled in the same way for all outcomes for the best-fitting models, parameter estimates were checked for similarity and then the posterior distributions were averaged across all models. Overall, there was an average of 142 (95% HDI: 124–167) counts of respiratory conditions for an average batch size of 1360 pigs, equating to 10.4% (95% HDI: 9.1–12.2%) prevalence. As would be expected, smaller on-farm batch sizes were associated with fewer respiratory condition counts. As batch size increased by 100 pigs, respiratory counts increased by a multiplicative factor of 1.051 (95% HDI: 1.046–1.057). However, the uncertainty around these estimates is very large, partly because we are lacking data from farms with large batch sizes (see Figure S2). We found that acquiring pigs from one batch source was associated with lower counts of respiratory conditions than acquiring from multiple farms (Fig. 3 and Table 3). There were no significant effects of either average number of days on farm (estimate: − 1.0027, 95% HDI: − 1.0086, 1.0033) or housing type (see Table S2) on respiratory counts. However, when comparing the slatted housing to the other housing types, although the highest density interval is wide (showing increased uncertainty in the estimate), the majority of the distribution sits above zero (Fig. 4), suggesting a probable higher count of respiratory conditions for slatted housing than for mixed housing, straw yards, or kennels.

**Table 3:** Summary of comparisons of the effect of the number of pig batch sources on the counts of respiratory conditions recorded at slaughter.

Comparison (no. of source farms)	Mean difference	95% highest density interval
1 vs 2	− 9.15	− 11.78, − 6.57
1 vs 3	− 9.36	− 13.06, − 5.81
1 vs > 3	− 9.79	− 13.93, − 5.87
2 vs 3	− 0.21	− 4.35, 3.83
2 vs > 3	− 0.63	− 5.14, 3.75
3 vs > 3	− 0.42	− 5.40, 4.35

### Respiratory effects on production outcomes

**Figure 5:**
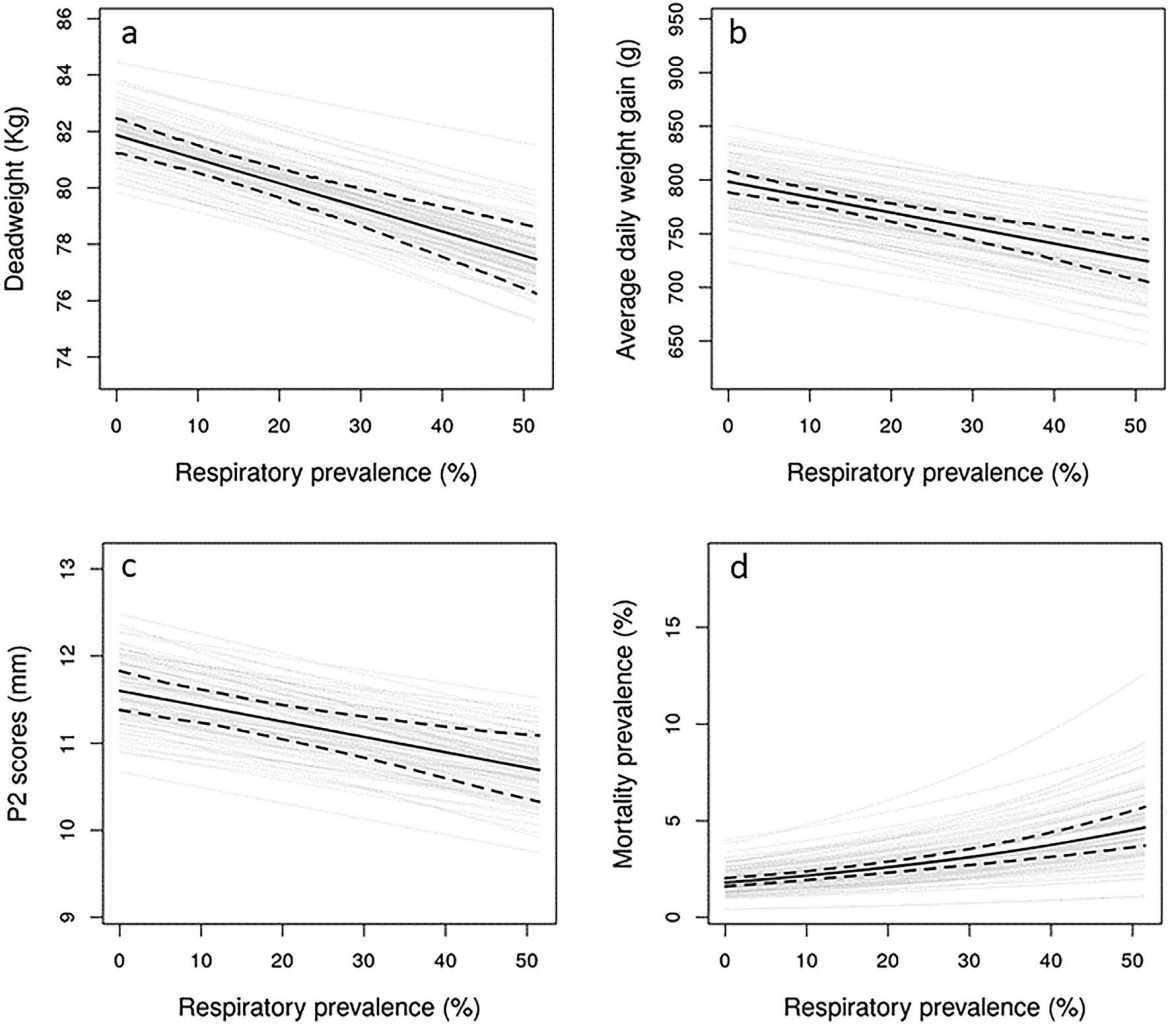
The effect of respiratory prevalence on (**a**) deadweight; (**b**) average daily weight gain; (**c**) P2 and (**d**) mortality. The posterior mean is denoted by the solid black line with the 95% highest density interval of the mean shown by the dashed lines. The grey lines show 100 representative samples from the posterior distribution marginalising across farm random effects. Respiratory prevalence was calculated from the counts of respiratory conditions and the total pigs in the batch.

For an average batch (with mean batch size and mean days on farm), the mean (HDI) production parameters were estimated as follows. Deadweight: 80.80 kg (95% HDI: 80.32, 81.28); average daily weight gain: 780 g (95% HDI: 772, 788); P2: 11.38 (95% HDI: 11.20, 11.57); mortality: 2.3% (HDI: 2.1, 2.5). Higher respiratory prevalence was associated with significantly poorer production performance for all production outcomes (Fig. 5). For every 1% increase in respiratory prevalence, we saw a 0.09 kg decrease in deadweight (95% HDI: − 0.12, − 0.06), a 1.44 g decrease in average daily weight gain (95% HDI: − 1.91, − 0.98) and a 0.018 mm decrease in P2 (95% HDI: − 0.03, − 0.009). A 1% increase in respiratory prevalence increased mortality by a multiplicative factor of 1.018 (95% HDI: 1.013, 1.023). This indicates, for an average batch, an increase of 5.76 counts of mortality for a 10% increase in respiratory prevalence (from 30.9 to 36.66).

### Additional deadweight predictors

Neither the number of batch sources, nor the housing type had a significant effect on deadweight (see Table S3 and Table S4).

### Additional average daily weight predictors

Average daily weight gain decreased by 32.35 g (95% HDI: − 35.51, − 29.19) for every week additional to the average number of weeks on farm, reflecting pig growth curves. This is to be expected as average daily weight gain decreases as pigs near slaughter weight and their growth curves plateau.

### Additional P2 predictors

**Figure 6:**
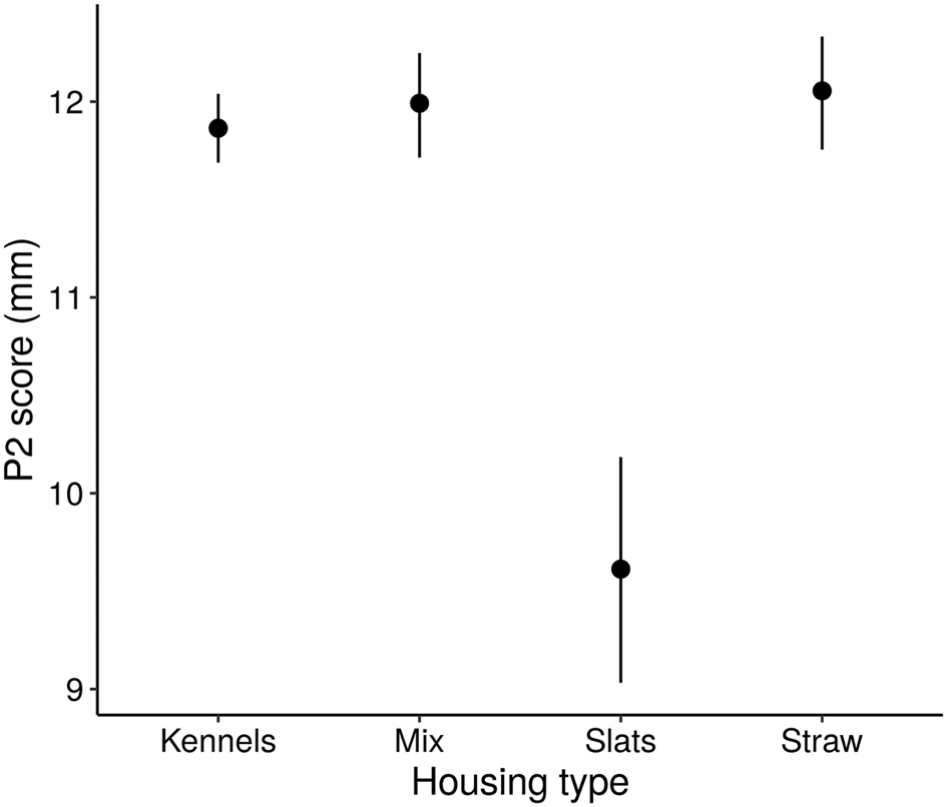
The effect of pig housing type on P2 score (a measure of back fat). Circles denote the mean posterior estimates and vertical line segments show their 95% highest density intervals. Kennels = kennelled housing; Mix = mixed housing (combinations of different housing types farm); Straw = straw yards and slats = slatted flooring.

Slatted floors were associated with lower P2 scores, with no difference in P2 between kennels, mixed housing and straw yards (Fig. 6 and Table 4). However, caution should be taken when interpreting these results as there were only 20 batches of pigs from three farms with slats. As expected, higher P2 scores were associated with higher average daily weight gain. For every 10 g increase in average daily weight gain, we saw a 0.05 increase in P2 (95% HDI: 0.04, 0.06). There was no significant effect of batch source on P2 (Table S5).

**Table 4:** Summary of comparisons of the effect of housing type on the backfat (P2) scores of batches of pigs.

Comparison	Mean difference	95% highest density interval
Slats vs straw yards	− 2.44	− 3.06, − 1.78
Slats vs kennels	− 2.25	− 2.83, − 1.65
Slats vs mixed	− 2.38	− 3.00, − 1.79
Kennels vs straw yards	− 0.19	− 0.52, 0.16
Kennels vs mixed	− 0.13	− 0.45, 0.20
Mixed vs straw yards	− 0.06	− 0.46, 0.32

### Additional mortality predictors

Mortality counts increased with increasing batch size by a multiplicative effect of 1.08 (95% HDI: 1.06, 1.09). For every additional week on farm, mortality increased by a multiplicative factor of 1.10 (95% HDI: 1.07, 1.12). Both results are logical in terms of increased probability of death given larger numbers of pigs and a longer time spent on farm.

## Discussion

In this study, we linked routinely recorded production and slaughter data with self-reported farm characteristics to assess the associations between farm characteristics, respiratory conditions and production outcomes. To guard against false positive findings, and to ameliorate the influences of noisy observational data, we took a hypothesis-driven, confirmatory approach by pre-registering the study methods and using Bayesian multi-level modelling and model selection using cross-validation. Using a Bayesian approach allowed us to quantify the uncertainty around our parameters by providing the probability of different parameter values given the data (see^41^).

The best-fitting Bayesian multi-level models suggested that, in all cases, farm characteristics indirectly influence production parameters through their effect on the prevalence of respiratory conditions. Additionally, farm characteristics directly influence the production parameters of deadweight and P2 scores (holding respiratory conditions constant). We found that a higher prevalence of respiratory conditions at slaughter was associated with pigs sourced from multiple origins and that higher prevalence of respiratory conditions detected at slaughter had negative associations with all production parameters of interest.

In line with our hypothesis and previous studies^14,17^, acquiring pigs from multiple source farms was associated with a higher prevalence of respiratory conditions at slaughter. This effect showed one source to be better than more than one source, with no difference in respiratory prevalence when acquiring from two, three, or more than three sources. Argostini et al.^42^ also found increased mortality for pigs sourced from multiple origins. We could not directly test for mortality effects as the most appropriate mortality model did not include a direct relationship with farm characteristics. Wiltshire (2018) reported that multi-phase pig production systems have higher risk of disease transmission in a realistic agent-based model^43^. The results of this study provide evidence that obtaining pigs from more than one source adversely affects respiratory health and productivity. This is likely, in part, to be associated with the differing pathogen and immune status of pigs from different sources, together with the potential stress associated with initial mixing pigs from different sources. All in/all out production batches have farm characteristics and health advantages over continuous systems as pigs are of a single age. However, the capacity of the all in/all out finishing farm may necessitate sourcing pigs from more than one breeding or nursery site to fill it over a short period of time.

We hypothesised that housing using straw would be associated with higher levels of respiratory illness. Findings from the literature concerning the effect of housing on respiratory conditions are mixed, with some reporting higher respiratory conditions in pigs on slats^17,20^ and others with pigs housed on straw-bedded flooring^22^. We hypothesised that straw may give increased respiratory conditions in view of the supporting literature, the fact that types of housing containing straw tend to have less precise and uniform control over the internal environment (e.g. curtains on barns versus an indoor thermostat system) and because straw can contribute to dust which can have adverse respiratory effects^36^. Regulation of internal environment, for example through more controlled ventilation, has been shown to be associated with lower respiratory condition prevalence^15–17^ but we found no significant effect of housing. However, there was a high level of uncertainty to the housing results, especially for slatted housing, represented by a large highest density interval (Fig. 3) and likely due to the small number of batches from three farms. Nevertheless, the majority of the parameter values contained within the pairwise intervals between slats and all other housing conditions were positive, meaning a higher prevalence of respiratory conditions in slatted systems was plausible and likely.

The number of days a batch of pigs spent on the finishing farm was included in the model to account for the assumption that pigs spending longer in production have more chance of contracting a respiratory disease. We did not find evidence to support this and these results suggest that it is not simply a case that additional days on farm leads to a greater chance of infection. However, in our dataset, the majority of batches were slaughtered within a similar timeframe (days of farm: mean = 94, sd = 10, min = 50, max = 129) and any additional time spent on farm outside of this may not be extreme enough to see an effect either on farm or at slaughter. Although we hypothesised that more days on farm would increase the risk of encountering a respiratory condition, it could also be the case that pigs spend longer on farm because of a respiratory condition. We are unable to disentangle these two data-generating processes in explaining the significant association between time on farm and respiratory prevalence because the former is a between-batch process where the latter is a within-batch process. Testing these hypotheses would require using within-batch, pig-level data (where all pigs within the batch arrive on the farm at the same number of days before slaughter weight) to assess whether pigs that contract respiratory conditions are kept longer on farm, or whether the association is driven by a between-batch time on farm effect. These hypotheses are not necessarily mutually exclusive. It could also be the case that the number of days on farm is an over-simplified proxy for a multitude of other unmeasured variables, such as diet, season, or earlier housing/management factors throughout the lifecycle. It would be worth future studies taking a causal modelling approach to build on these results.

Increased prevalence of respiratory disease at slaughter was associated with higher mortality, lower deadweight, lower average daily weight gain and lower back fat (P2). Our findings for back fat oppose two other studies which found no effect of respiratory conditions^6^ or lung lesions^44^ on fat depth scores. Our results for mortality, deadweight and average daily weight gain mirror results from previous, smaller, but potentially more sensitive studies. For example, two studies using the national British Pig Health Scheme (BPHS) dataset found that increases in measures of enzootic pneumonia and pleurisy were associated with lower carcass weights^12,26^. A New Zealand study detected reductions of 2.2 g in average daily weight gain, for each 1% of lung volume affected by enzootic pneumonia^25^ and Straw et al.^28^, in a small scale study, also found a decrease in average daily weight gain when pigs were exposed to causative agents of pneumonia. A study of naturally occurring PRRSV and influenza Type A co-infection challenges on US pig farms found both a reduction in average daily weight gain and an increase in mortality as the disease prevalence increased^6^. The CCIR data, which we used as health data, has been criticised for being relevant across farms, but not at the batch level, due to issues of incorrect identification of pigs in batches^32^ and a lack of sensitivity for some certain conditions^33^. However, we show here that CCIR data may be useful as a surveillance tool at batch level for broad categories of health conditions, mirroring relationships between key production traits and respiratory conditions seen in previous studies.

Both the models for P2 and deadweight allowed for direct links between farm characteristics and the production outcomes. Of these, we found that housing had a significant affect on P2, with slats associated with lower P2 scores. However, the effect of slats on P2, as well as the effect of slats on respiratory prevalence (discussed above) are likely to be conflated with other factors. Only 20 batches from three farms were housed on slats. Thus, the effects of slats may instead reflect other unknown specifics of those farms or companies and may not be applicable to the wider population. Secondly, the genetics of outdoor-bred pigs (those finished on straw in our dataset) mean these breeds tend to be less lean, which could impact P2 results. We therefore stress the tentative nature with which we present our results for slatted systems and highlight the need for more farms, from more companies, with slats in future analyses.

In order to support implementation of sustainable improvements in production, particularly where these require significant financial and other resources, good evidence and better mechanistic understanding of the factors affecting health and production performance in pigs is vital. Interestingly, our model comparison approach highlighted that the four production traits were not all best modelled in the same way and this is something for future studies to take into consideration. There are additional relevant associations to be explored through this analytical approach, if the data can be obtained. Examples of these include exploring the association of respiratory conditions at slaughter with herd status for different pathogens (or combinations of pathogens) or with herds with different levels of antimicrobial use. Additionally, possible future analyses could include finer level details by, for example, modelling the relationship between specific respiratory conditions first (e.g. using latent variable models^45^) rather than using simple sum scores as used in this study. This would be of use given the previous studies have identified different risk factors for different respiratory conditions (e.g.^17^). Specific hypotheses about the transmission and development of respiratory conditions and their detection at slaughter could also be tested using formal mathematical models, such as agent-based models of livestock production networks (e.g. see^43^). Any future studies would be strengthened by obtaining data from a wider pool of producers, providing more varied data on management practices and infrastructure and allowing for analysis of the effects of disinfection and ventilation. Finally, future analyses would benefit from a more standardised data recording format within the pig industry for farm characteristics and batch level production data. Standardised recording would allow for useful analyses with nationally held health and welfare datasets and for comparisons of the production impacts of differing farm characteristics protocols.

## Conclusions

In summary, we found that acquiring pigs from multiple sources was linked to a higher prevalence of respiratory conditions at slaughter. Increased prevalence of respiratory disease was associated with poorer farm-based performance metrics of mortality, deadweight, back fat and pig carcass weight. Our results show that carcass inspection data recorded in the abattoir are a valuable tool for monitoring respiratory conditions at batch-level. We advocate standardised recording of data in the pig industry to better understand how farm characteristics impact on production performance and health, to enable investigation and monitoring of sustainable farming practices in the future.

## Supplementary Information

Supplementary Information 1.

Supplementary Information 2.
