# Supplementary material for: Modelling the links between farm characteristics, respiratory health and pig production traits
Source: Sci Rep. 2021 Jul 2;11:13789. doi: 10.1038/s41598-021-93027-9 (PMC8253804; doi:10.1038/s41598-021-93027-9)
Supplement: Supplementary file 1 — Supplementary Information 1. [file 41598_2021_93027_MOESM1_ESM.pdf]

1. Farm characteristics

Farm name:	
Contact person:	
	Farm owner / manager / herdsperson (delete as appropriate)
Farm postcode:	
Email:	
CPH Number (s):	
Farm assurance	Red Tractor – Number:<br>Other (please state):<br>None:
Herdmark:	
Private vet name:	Specialist pig vet? Yes ☐ No ☐
Staff onsite:	Number full-time: Number part-time:

1.1. How many pigs in total are kept on this site? ....

1.2. What is the type of production on the farm?

**Production type**		**Yes**	**No**
**<u>Breeder</u> unit**			
**<u>Farrow-to-finish</u> unit**			
**<u>Weaning to finishing</u> unit**			
Over the last year, on average, each batch was supplied by:	Single breeding unit		
	2 breeding units		
	3 breeding units		
	>3 breeding units		
**<u>Grower to finishing</u> unit**			
Over the last year, on average, each batch was supplied by:	Single weaner unit		
	2 weaner units		
	3 weaner units		
	>3 weaner units		

1.3. Select the closest description of the ***predominant*** type of accommodation for each applicable stage of production (please tick only one box for each age-group).

Accommodation type	Dry Sows	Weaners	Growers	Finishers
Fully slatted				
Part slatted				
Kennel (with bedding) + ***indoor*** run				
Kennel (with bedding) + ***outdoor*** run				
Kennel (no bedding) + ***indoor*** run				
Kennel (no bedding) + ***outdoor*** run				
Straw yards				
Outdoor				
Other (please specify below)				

1.4. What age is the predominant type of accommodation for each stage and number of pig places in each stage (leave box blank if stage not present on your site):

Accommodation type	Dry Sows	Farrowing	Weaners	Growers	Finishers
Age (years)					
Number of pig places					

1.5. Tick type of ventilation in use in the predominant type of accommodation for each stage (leave box blank if stage not present on your site):

Feature	Dry Sows	Farrowing	Weaners	Growers	Finishers
Assisted ventilation					
Natural ventilation					
Other, please specify:					

1.6. Do you have an All-in All-out (AIAO) system currently operating on the site?

Yes ☐ No ☐

1.6.1. If an AIAO system is used: How is it implemented (please tick appropriate boxes):

	**Farrowing**	**Weaners**	**Growers**	**Finishers**
AIAO by pen				
AIAO by room				
AIAO by building				
AIAO by paddock				
Approximate downtime (days) between pigs leaving and next batch entering				
Wash down between batches?				
Disinfection between batches?				

1.6.2. Feeding regime (please tick appropriate box):

	**Dry feed**	**Wet feed**	**Home - Mix**	**Purchased Compound**	**By-product**
Dry sows					
Farrowing					
Weaners					
Growers					
Finishers					

2. Health/Disease Questions

2.1. Have you had any of the following types of disease problem at any time on the farm in the two-year period from January 2016 to December 2017?

**Disease description**	**In 2016-17** *please tick **one** box*			**What type of pigs were most affected in 2016-17** *please tick **all that apply***			
	**Not seen**	**Minor problem**	**Main or sometimes major problem**	**Sucking pigs**	**Weaner/ growers**	**Finishers**	**Adult breeding pigs**
2.1.1 Diarrhoea/ scour							
2.1.2 Respiratory disease e.g. Coughing							
2.1.3 Nervous signs e.g. Meningitis-type							
2.1.4 Lameness							
2.1.5 Wasting							
2.1.6 Skin disease							
2.1.7 Sudden or rapid deaths							
2.1.8 Reproductive problems							
2.1.9 Other (please specify)							

2.2. What vaccines have been used in pigs on your unit in 2016 and 2017?

**Vaccine**	**Rearing pigs** *please tick*	**Breeding pigs** *please tick*
Enzootic pneumonia *(Mycoplasma hyopneumoniae)*		
PRRS virus (blue ear disease)		
PCV2 (porcine circovirus – PMWS)		
Glässer’s disease (*Haemophilus parasuis)*		
Erysipelas		
Porcine parvovirus (PPV)		
Pleuropneumonia *(Actinobacillus pleuropneumoniae)*		
Streptococcus suis		
E. coli (for diarrhoea)		
E. coli (for oedema disease)		
Clostridial disease (for piglet diarrhoea)		
Clostridial disease (for sows – sudden death)		
Leptospirosis		
Swine influenza		
Salmonella		
Lawsonia (ileitis)		
Other (please state)		

2.3. Do you know which of the following specific diseases have caused clinical signs or deaths in pigs on your farm in 2016 and/or 2017?

** Pig age groups:*

1. Suckling pigs **2.** Weaner – growers **3.** Finishers **4.** Adults

Cause	Disease	Disease not seen *Please tick*	Disease occurrence suspected *Please tick*	Disease confirmed by vet *Please tick*	Don't know *Please tick*	What age* of pig was affected/most affected
**RESPIRATORY DISEASE**						
2.3.1 Mycoplasma hyopneumoniae	**Enzootic pneumonia**					
2.3.2 PRRS virus	**PRRS**					
2.3.3 Swine influenza virus	**Swine influenza**					
2.3.4 Pasteurella multocida	**Pneumonia due to pasteurellosis**					
2.3.5 Actinobacillus pleuropneumoniae	**Pleuropneumonia**					
2.3.6	**Other (please state)**					
**GENERALISED**						
2.3.7 Streptococcus suis	**Strep septicaemia (inc meningitis/lameness)**					
2.3.8 Haemophilus parasuis	**Glässer's disease**					
2.3.9 Erysipelothrix rhusiopathiae	**Erysipelas**					
2.3.10 Porcine circovirus 2 (PCV-2)	**PMWS and PCVAD (wasting disease)**					
2.3.11 PCV2-related	**PDNS**					
2.3.12	**Other (please state)**					
**NERVOUS DISEASE (Strep suis and Glassers included above)**						
2.3.13 E. coli	**Oedema disease/bowel oedema**					
2.3.14	**Other (please state)**					

2.3. continued from previous page

Cause	Disease	**Not present** Please tick	**Disease occurrence suspected** Please tick	**Disease confirmed by vet** Please tick	**Don't know** Please tick	**What age* of pig was affected/most affected**
**GUT DISEASE**						
2.3.15 Clost. perfringens	**Clostridial enteritis**					
2.3.16 Isospora suis	**Coccidiosis**					
2.3.17 E. coli	**E coli diarrhoea**					
2.3.18 Rotavirus	**Rotavirus diarrhoea**					
2.3.19 Salmonella serotypes	**Clinical salmonellosis**					
2.3.20 Lawsonia	**Ileitis**					
2.3.21 Brachyspira hyodysenteriae	**Swine dysentery**					
2.3.22 Brachyspira pilosicoli	**Pilosicoli colitis**					
2.3.23 Ascaris suum (roundworm)	**Milk spot**					
2.3.24	**Intestinal torsions**					
2.3.25	**Other (please state)**					
**SKIN DISEASE**						
2.3.26 Sarcoptes mite	**Mange**					
2.3.27 Staphylococcus hyicus	**Greasy pig**					
2.3.28	**Other (please state)**					
**LAMENESS (Strep suis and Glassers included above)**						
2.3.29 Mycoplasma hyosynoviae	**Mycoplasmal arthritis**					
2.3.30 OCD	**Osteochondrosis**					
2.3.31	**Other (please state)**					

Thank you for taking your time to provide this valuable information!
