# Supplementary material for: Modelling the links between farm characteristics, respiratory health and pig production traits
Source: Sci Rep. 2021 Jul 2;11:13789. doi: 10.1038/s41598-021-93027-9 (PMC8253804; doi:10.1038/s41598-021-93027-9)
Supplement: Supplementary file 2 — Supplementary Information 2. [file 41598_2021_93027_MOESM2_ESM.pdf]

# Modelling the links between farm characteristics, respiratory health and pig production traits.

Gray, H[1]. Friel, M[1]. Goold, C[1]. Smith, R. P[2]. Williamson, S. M.[3]. Collins, L. M.[1*]

[1] School of Biology, Faculty of Biological Sciences, University of Leeds, United Kingdom

[2] Animal and Plant Health Agency (APHA), Epidemiology Department, Addlestone, United Kingdom

[3] APHA, Rougham Hill, Bury St Edmunds, Suffolk, United Kingdom

*Corresponding author: l.collins@leeds.ac.uk

## Supplementary Material

Table S1. Sensitivity analysis for selecting the slaughter range for matching the production data to the CCIR data. When using 5 weeks before the production slaughter date and 4 weeks after the production slaughter date, we achieve the best matching in the CCIR data.

| Weeks before slaughter date | Weeks after slaughter date | Zero matches | | Too few pigs matched | | Correct matches | | Too many pigs matched | | Total Batches |
|---|---|---|---|---|---|---|---|---|---|---|
| | | No. batches | % | No. batches | % | No. batches | % | No. batches | % | |
| 3 | 3 | 82 | 8 | 350 | 34 | 582 | 57 | 3 | <1 | 1017 |
| 3 | 4 | 82 | 8 | 337 | 33 | 591 | 58 | 4 | <1 | 1014 |
| 3 | 5 | 82 | 8 | 334 | 33 | 590 | 58 | 6 | <1 | 1012 |
| 4 | 3 | 81 | 8 | 257 | 25 | 674 | 66 | 3 | <1 | 1015 |
| 4 | 4 | 81 | 8 | 248 | 25 | 678 | 67 | 5 | <1 | 1012 |
| 4 | 5 | 81 | 8 | 247 | 24 | 676 | 67 | 6 | <1 | 1010 |
| 5 | 3 | 81 | 8 | 235 | 23 | 696 | 69 | 3 | <1 | 1015 |
| **5** | **4** | **81** | **8** | **227** | **22** | **699** | **69** | **5** | **<1** | **1012** |
| 5 | 5 | 81 | 8 | 226 | 22 | 697 | 69 | 6 | <1 | 1010 |

S1 Questionnaire: see separate document

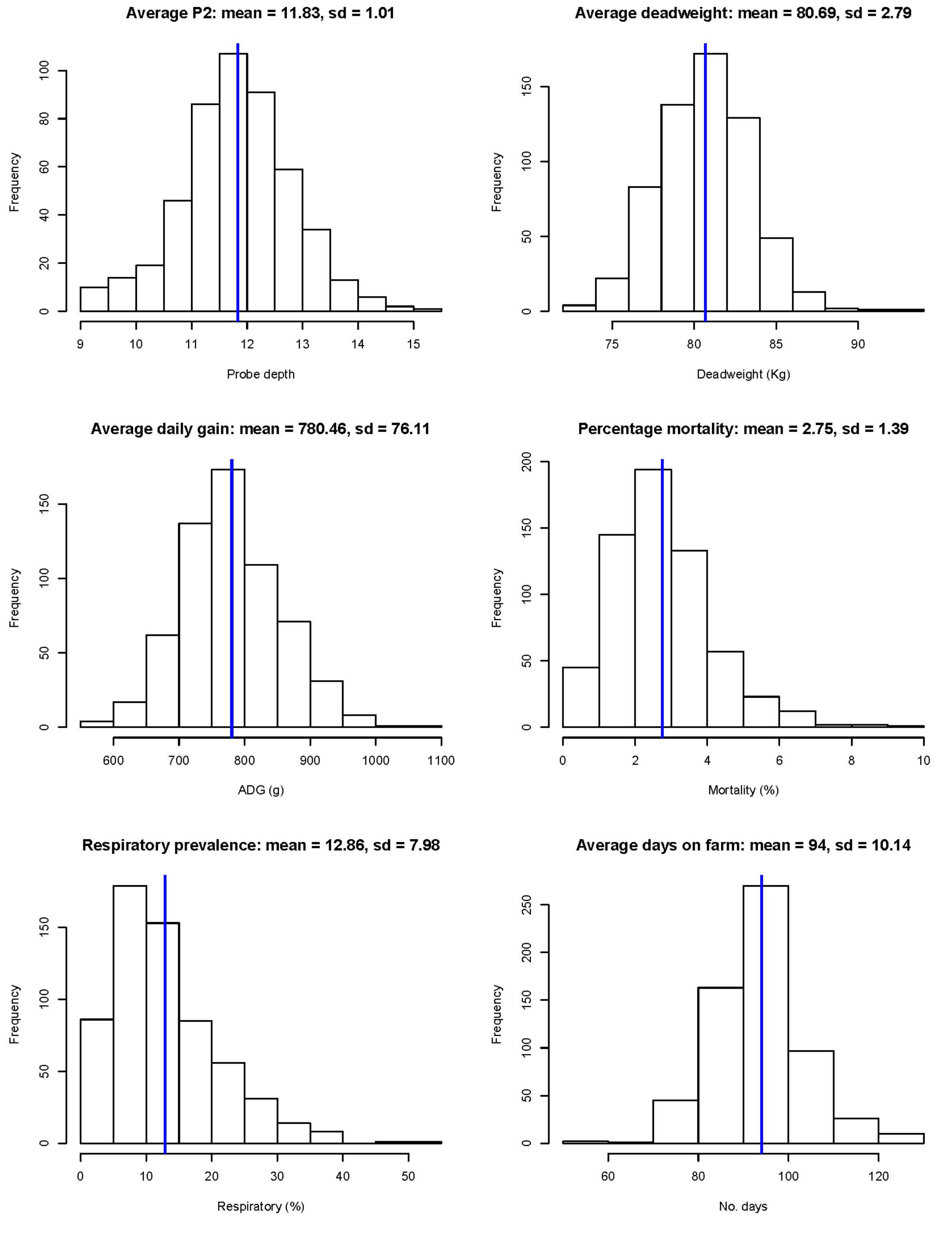


Figure S1. Histograms of raw data, showing overall distributions with the mean denoted by the blue line.

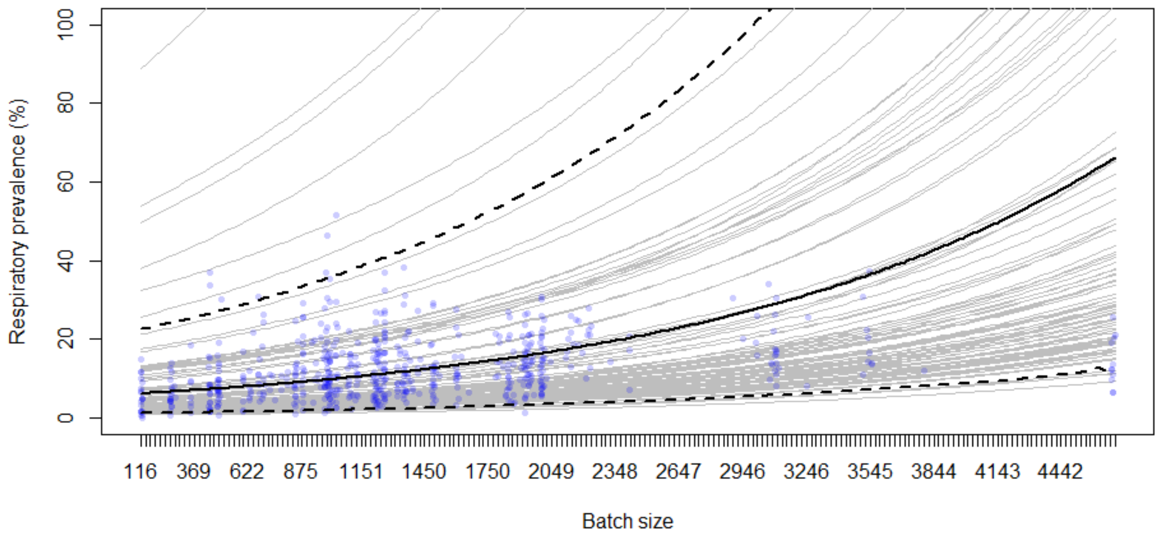


Figure S2. The effect of batch size on respiratory prevalence. The posterior mean is denoted by the solid black line with the 95% highest density interval of the mean shown by the dashed lines. The grey lines show 100 representative samples from the posterior distribution marginalising across farm random effects. Respiratory prevalence was calculated from the counts of respiratory conditions and the total pigs in the batch. Raw data are shown by blue dots and highlight the paucity of farms with very large batch sizes ( > 2000 pigs).

Table S2. Summary of comparisons of the effect of housing type on counts of respiratory conditions recorded at slaughter

| Comparison (housing type) | Mean difference | Highest density interval |
|---|---|---|
| Slats vs straw yards | 4.84 | -1.79, 12.48 |
| Slats vs kennels | 5.04 | -1.47, 12.20 |
| Slats vs mixed | 6.02 | -0.31, 13.17 |
| Kennels vs mixed | 0.98 | -1.47, 3.38 |
| Kennels vs straw yards | -0.19 | -3.18, 2.62 |
| Mixed vs straw yards | -1.18 | -4.30, 2.11 |

Table S3. Summary of comparisons of the effect of the number of pig batch sources on the deadweight of batches of pigs.

| Comparison (no. of source farms) | Mean difference | Highest density interval |
|---|---|---|
| 1 vs 2 | 0.52 | -0.36, 1.44 |
| 1 vs 3 | 0.99 | 0.030, 1.97 |
| 1 vs >3 | 0.76 | -0.27, 1.81 |
| 2 vs 3 | 0.47 | -0.39, 1.30 |
| 2 vs >3 | 0.24 | -0.64, 1.09 |
| 3 vs >3 | -0.23 | -1.19, 0.75 |

Table S4. Summary of comparisons of the effect of housing type on deadweight of batches of pigs.

| Comparison (housing type) | Mean difference | Highest density interval |
|---|---|---|
| Slats vs straw yards | 1.03 | -0.57, 2.85 |
| Slats vs kennels | 0.67 | -0.92, 2.24 |
| Slats vs mixed | 1.01 | -0.60, 2.68 |
| Kennels vs mixed | 0.34 | -0.41, 1.10 |
| Kennels vs straw yards | 0.37 | -0.45, 1.21 |
| Mixed vs straw yards | 0.025 | -0.99, 0.93 |

Table S5. Summary of comparisons of the effect of the number of pig batch sources on the backfat (P2) of batches of pigs.

| Comparison (no. of source farms) | Mean difference | Highest density interval |
|---|---|---|
| 1 vs 2 | 0.15 | -0.23, 0.53 |
| 1 vs 3 | 0.11 | -0.30, 0.52 |
| 1 vs >3 | -0.12 | -0.55, 0.31 |
| 2 vs 3 | -0.04 | -0.39, 0.29 |
| 2 vs >3 | -0.27 | -0.61, 0.11 |
| 3 vs >3 | -0.22 | -0.64, 0.17 |
